# Superior Mesenteric Artery Pseudoaneurysm Induced by Accidental Ingestion of a Foreign Body: Case Report

**DOI:** 10.1016/j.ejvsvf.2022.01.002

**Published:** 2022-01-10

**Authors:** Ahmed Al Harthy, Alexandre Belot, Patrick Feugier

**Affiliations:** a Service de Chirurgie Vasculaire et Endovasculaire, Hôpital Lyon Sud, Hospices Civils de Lyon, Lyon, France; b Service de Néphrologie–Rhumatologie–Dermatologie Pédiatriques, Hôpital Femme Mère Enfant, Hospices Civils de Lyon, Lyon, France

**Keywords:** Foreign body, Paediatric, Superior mesenteric artery, Superior mesenteric artery pseudoaneurysm

## Abstract

**Background:**

Superior mesenteric artery (SMA) pseudoaneurysm is a very rare condition, typically associated with trauma, inflammation, and infection, and as a post-operative complication. If left untreated it can lead to serious consequences such as rupture and fatal haemorrhage.

**Report:**

A 17 year old male presented to the emergency department with a history of intermittent progressive epigastric pain with no preceding significant symptoms of a possible cause. He was initially treated conservatively until the intensity of pain was so severe an abdominal computed tomography (CT) scan was justified. A pseudoaneurysm of the SMA was found. Full inflammatory and immunological workup was unremarkable. Repeat CT scan showed the SMA pseudoaneurysm was larger, mandating surgical intervention; the vascular surgeon suggested an exploratory laparotomy. Intra-operatively, unexpectedly, a wooden foreign body measuring 5.0 × 0.3 × 0.5 cm was seen once the aneurysm sac was opened. The pseudoaneurysm was repaired and the abdomen closed after ascertaining that all other organs were intact. The patient had a simple recovery with no complications and was discharged home. The follow up CT scans were unremarkable.

**Conclusion:**

Pseudoaneurysm of the SMA in the paediatric age group is an extremely rare and life threatening phenomenon. The clinical presentation may be subtle, leading to delayed diagnosis. Early surgical intervention may be lifesaving and prevent further complications.

## Highlights

• Pseudoaneurysm due to swallowed foreign body is an extremely rare pathology.
• Visceral artery pseudoaneurysm are usually silent but potentially fatal.
• Surgical intervention is mainstay treatment of visceral artery pseudoaneurysm.

## Introduction

Visceral artery aneurysms and pseudoaneurysms (VAPA) are rare pathological conditions with an estimated prevalence of 0.1%–2%,^1^ and the mortality rate due to aneurysm rupture and massive bleeding is reported to range from 25% to 80%.^2^ According to the European Society of Vascular Surgery guidelines, intervention should be considered for asymptomatic true mesenteric aneurysms of ≥25 mm in length and asymptomatic pseudoaneurysms irrespective of size; urgent repair is recommended for symptomatic patients, irrespective of size or location.^1^

The present study is the case report of an adolescent who presented with a superior mesenteric artery (SMA) pseudoaneurysm induced by an ingested foreign body, which was managed surgically.

## Case report

A 17 year old male with no significant past medical history presented to the emergency department (ED) with a sudden onset of severe abdominal pain located mainly in the epigastric region radiating to the back, which was associated with nausea and vomiting. During examination, there was generalised tenderness on deep palpation of the abdomen. There was no guarding or rigidity, and McBurney's sign was negative. Blood investigations and abdominal ultrasound were unremarkable. Therefore, the patient was sent home once his pain was alleviated by analgesics.

**Figure 1:**
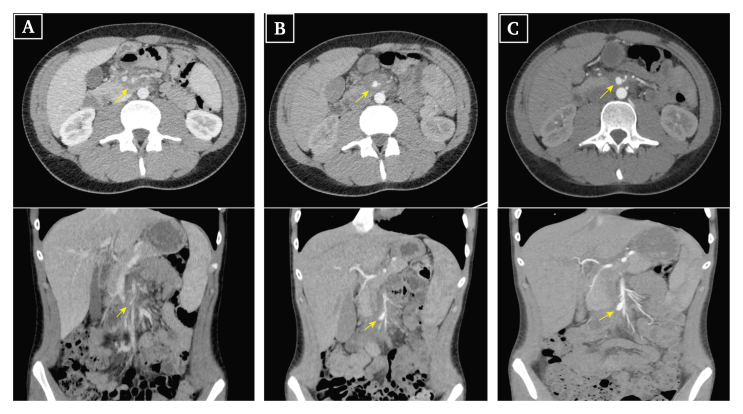
Computed tomography angiography of the superior mesenteric artery in axial and coronal sections showing the evolution of the pseudoaneurysm (yellow arrow) on (A) day 0, (B) day 2, and (C) day 7 of the hospital stay.

One week later, the patient re-presented to the ED with a similar presentation. On questioning, he denied being pain free since his previous visit to the hospital. Blood tests found a slightly elevated C reactive protein (CRP; 42 mg/L), the results of other tests were normal, including neutrophil count (3.4 g/L). An abdominal computed tomography (CT) scan with intravenous (IV) and oral contrast was performed, which revealed abnormal fat infiltration at the root of the mesentery with sub-centimetre lymphadenopathy with non-specific features. The SMA had an irregular demarcation, and radiological features were suggestive of a possible SMA pseudoaneurysm (Fig. 1A; Supplementary Video S1).

Supplementary video related to this article can be found at https://doi.org/10.1016/j.ejvsvf.2022.01.002

The following is the supplementary data related to this article:

Video S1

The patient was hospitalised in the paediatric ward for close surveillance and to complete the diagnostic workup. On admission, he developed a high grade fever (39 °C) and therefore broad spectrum antibiotic therapy (ceftriaxone 2 g IV once daily) was initiated. A full septic workup was unremarkable except for an elevated CRP (83 mg/L). Serial blood workup found a progressive decrease in haemoglobin from 11.2 g/dL to 8.7 g/dL within 24 hours; thus, an abdominal CT scan was repeated on day 2 post-admission, which found a significant SMA pseudoaneurysm, the greatest diameter of which was 7 mm on axial section and 15 × 4 mm on coronal section, associated with abnormal infiltration of the mesentery around the aneurysm sac (Fig. 1B; Supplementary Video S2).

Supplementary video related to this article can be found at https://doi.org/10.1016/j.ejvsvf.2022.01.002

The following is the supplementary data related to this article:

Video S2

The case was discussed in a multidisciplinary meeting where various differential diagnoses were proposed, including vasculitis like polyarteritis nodosa, septic pseudoaneurysm of the SMA, and inflammatory bowel disease. It was therefore decided to initiate corticosteroid therapy until a diagnosis was established, and to conduct extensive immunological and infectious screening (hepatitis B and C, adenovirus DNA, cytomegalovirus DNA, antinuclear cytoplasmic antibodies, anti-DNA antibodies, antichromatin antibodies, and complement screening), which were all negative.

The abdominal CT angiogram was repeated on day 7 of admission and showed an increase in the size of the SMA pseudoaneurysm to 8 mm on axial section and 20 × 8 mm on coronal section (Fig. 1C; Supplementary Video S3). The opinion of the vascular surgeons was sought, and the advice was for an urgent exploratory laparotomy to be performed.

Supplementary video related to this article can be found at https://doi.org/10.1016/j.ejvsvf.2022.01.002

The following is the supplementary data related to this article:

Video S3

Under general anaesthesia, a midline laparotomy was carried out and the greater sac of the peritoneum was explored. No blood collection or effusion was found. Manual exploration of the abdominal cavity revealed no gross anomaly. The SMA was controlled and once an incision into the aneurysm sac was made, an object in the form of a stick, 1–2 mm thick and 4 cm long was retrieved. Samples were sent for histopathology examination and bacterial culture. After extraction of the foreign body, part of the arterial wall border was damaged and thus the artery was repaired using autologous great saphenous vein as a patch. Bowel exploration was normal. Abdominal lavage was performed before closure. Antibiotic therapy was initiated (cefotaxime 2 g IV three times daily, metronidazole 500 mg orally [PO] three times daily, and gentamicin 200 mg PO once daily).

**Figure 2:**
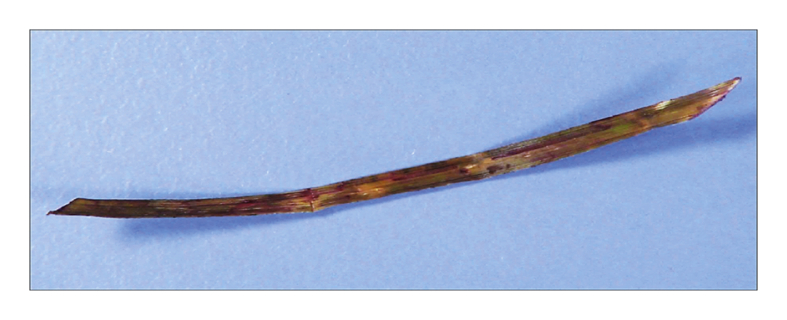
A piece of wood extracted from within the superior mesenteric artery pseudoaneurysm.

Histopathology examination reported a foreign body of vegetable origin (a piece of wood) measuring 5.0 × 0.3 × 0.5 cm (Fig. 2). After presenting the patient and his family with the findings of the surgery, they recalled that the patient had swum 10 years earlier in a lake surrounded by many small trees with branches similar to the one removed from his artery.

Bacterial culture of the aneurysm sac isolated three species of bacteria (*Streptococcus anginosus*, *Aggregatibacter aphrophilus*, and *Enterobacter cloacae*). The antibiotic regimen was modified according to the bacterial sensitivities to ceftriaxone 2 g IV once daily and levofloxacin 500 mg orally twice daily, which was continued for one month. The patient made a good recovery. A follow up abdominal CT scan done five days post-operatively found no signs of intraperitoneal collection or abscess formation, and with good SMA patency. He was discharged home on day 8 post-operatively in a stable condition. The patient was seen on a follow up visit after five months, with the CT angiogram showing an intact SMA arterial wall with no aneurysm recurrence. The patient was pain free.

## Discussion

Pseudoaneurysms due to swallowed foreign bodies are rare. The aortic arch has been found to be the most commonly affected site.^3^ Other cases have been reported in the descending aorta, the left common carotid artery,^3^ the right subclavian artery, the left subclavian artery,^4^ and the external carotid artery.^5^ VAPAs are extremely rare in vascular surgery practice; the majority of cases occur secondary to pancreatitis or trauma.^6^ Other possible causes are iatrogenic, idiopathic, subsequent to an arterial dissection, infective endocarditis, and uncontrolled hypertension.^7^^,^^8^

SMA aneurysms and pseudoaneurysms typically occur on the proximal 5 cm of the SMA.

Most patients are asymptomatic, but some may present with abdominal pain, nausea, and vomiting. SMA aneurysms are usually found incidentally when performing abdominal imaging for other reasons.^1^

In most cases, surgeons opt to repair pseudoaneurysms because of the high mortality rate if left untreated. Complications associated with VAPAs include rupture, acute thrombosis, or embolisation to small bowel vessels.^9^ Various surgical techniques have been described for the management of SMA pseudoaneurysms:^10^ aneurysmorrhaphy, bypass, and exclusion constitute the conventional surgical treatment; and, although there are very few cases of endovascular management due to the rarity of the disease, stent graft exclusion and coil embolisation have been described.^9^

In this case, the patient presented ambiguously with recurrent abdominal pain of increasing intensity and high grade fever. Serial abdominal CT scans revealed an enlarging SMA pseudoaneurysm, which justified exploratory laparotomy with pseudoaneurysm repair. On opening the aneurysm sac, a wooden foreign body was found, which explained the clinical manifestation. The hypothesis is that the patient accidentally ingested the foreign body, which then descended the gastrointestinal tract to the stomach/small bowel traversing its wall towards the SMA, eventually settling within the arterial wall, and, over a period of time, evolving into a localised infection leading to pseudoaneurysm formation. However, it cannot be excluded that chemical or mechanical arterial damage caused by the foreign body may also have contributed to the pathological condition.

## Conflict of interest

None.

## Funding

None.
